# Effects of Nanocellulose on the Physicochemical‐Digestive Characteristics of Corn Starch

**DOI:** 10.1002/fsn3.72083

**Published:** 2026-07-06

**Authors:** Yanyan Liu, Shuyao Li, Junjie Yuan, Huiying Zhang, Xueli Gao, Yonghui Wang, Shenghua He, Weiyun Guo, Guanghui Li

**Affiliations:** ^1^ Medical College Xuchang University Xuchang China; ^2^ Collaborative Innovation Center of Functional Food by Green Manufacturing Xuchang Henan Province China; ^3^ Food and Pharmacy College Xuchang University Xuchang China

**Keywords:** corn starch, digestive properties, microstructure, nanocellulose

## Abstract

This study investigated the effects of nanocellulose (NC) on the physicochemical properties, multi‐scale structure, and in vitro digestibility of corn starch (CS). The results showed that NC complexed with CS through non‐covalent bonds, and the absorbance ratio of 1047 to 1022 cm^−1^ increased from 0.38 to 0.72 with increasing NC content. NC‐CS complexes exhibited an A + V‐type structure with relative crystallinity ranging from 26.75% to 29.05%. Scanning electron microscopy demonstrated that NC‐CS complexes possessed thin and smooth surfaces. The onset temperature, peak temperature, and conclusion temperature of CS were increased after complexation with NC, while the enthalpy showed no significant change. Moreover, the incorporation of NC resulted in increased solubility and transparency but decreased swelling power of CS. Notably, the slowly digestible starch (SDS) content increased from 32.01% to 58.07%, and CS with 5% NC exhibited the highest SDS content. The sum of SDS and resistant starch (RS) also increased (58.74%–69.73%), leading to reduced starch digestibility. In all, the changes in physicochemical properties and multi‐scale structure of CS induced by NC resulted in decreased in vitro digestibility. This study provides foundations for the development of low glycemic index starch‐based foods using NC.

## Graphical abstract

Hydrogen bonding interacted between CS and NC. NC‐CS complexes exhibited thinner and smoother surfaces. The digestion of NC‐CS complexes decreased due to the increased SDS. The gelatinization temperatures of CS were increased after complexation with NC.

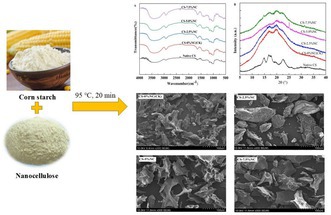

## 1 Introduction

Starch, the main source of dietary energy for humans, is derived from various plants such as cereals, roots, and fruits. Corn starch (CS), the most widely produced starch in China, is commonly used as a thickener and texture modifier in the food industry. However, due to its inherent drawbacks‐including inadequate shear resistance, thermal instability, susceptibility to retrogradation, and rapid digestibility, CS has limited broader application in the food industry. In order to improve the properties of CS, physical (e.g., annealing, microwave, heat moisture treatment), chemical (e.g., acid hydrolysis, esterification, oxidation) and biological (e.g., α‐Amylase, β‐amylase, glucoamylase) modification strategies have been employed (Obadi et al. 2023). With the growing demand for healthy foods, natural components such as polysaccharide, polyphenols, and protein have been used to enhance the physiochemical properties and in vitro digestibility of CS. For instance, Li, Duan, et al. (2026) reported that guar gum increased the pasting properties and decreased the thermal stability of CS. Luo et al. (2025) demonstrated that the thermal stability, viscoelastic, gelatinization and retrogradation of the CS‐pectin/konjac glucomannan complexes were improved by the partial gelatinization treatment. Oladele et al. (2019) found that phenolic extracts from grape pomace and sorghum bran increased the peak viscosity and enthalpy of maize starch, and complexes of starch and phenolic compounds were formed through hydrogen and other bonds.

Dietary fiber, a type of non‐starch polysaccharide derived from plant cell walls, is recognized for its health benefits, including hypoglycemic, hypolipidemic, antioxidant, and prebiotic activities (Li et al. 2024). Consequently, dietary fiber has been applied in various foods such as meat, bread, dairy, and snack (Ermis et al. 2026). Numerous studies have reported that dietary fiber affected the physicochemical properties and digestibility of starch by altering the multiscale structure and inhibiting the enzyme activity (Chen et al. 2025; Li et al. 2025; Lv et al. 2025). Gu et al. (2026) showed that soluble dietary fibers extracted from mung bean hull inhibited starch gelatinization, increased ordered structure, and thereby delayed the digestion of starch. Lv et al. (2025) found that cellulose, hemicellulose, and lignin enhanced thermal stability and reduced viscosity and digestibility of cassava starch. Xu et al. (2025) observed that soluble dietary fiber from large‐leaf yellow tea inhibited the short‐term retrogradation, reduced crystallinity and short‐range order of buckwheat starch.

Recently, nanocellulose (NC) has attracted more and more attention due to its excellent biocompatibility, transparency, water absorption, and unique rheological properties. NC is prepared from plant, animal or bacterial using chemical, mechanical, enzymatic, and other methods (Fan et al. 2026). The applications of NC in edible films and Pickering emulsion have been widely reported (de Almeida et al. 2024; de Oliveira et al. 2025; Li, Ma, et al. 2026; Mary et al. 2025; Zou et al. 2025). Moreover, the utilization of NC in foods has also been reported (Fan et al. 2026). For instance, Cunha et al. (2024) demonstrated that nanofibrillated improved the consistency and reduced the syneresis of yogurt. Zhao et al. (2025) found that treatment with the arabinoxylan‐10% nanofibrillated cellulose composite resulted in the highest gel strength, enhanced short‐range order, and reduced digestibility of starch.

Currently, the effect of NC on the physicochemical properties and digestibility of starch was rarely explored. Therefore, the NC‐CS complexes were prepared through co‐gelatinization, and the structural, physicochemical properties, and in vitro digestibility of NC‐CS complexes were investigated. This study offers new insights for developing functional starch‐based foods with a lower glycemic index (GI) using NC.

## 2 Methods and Materials

### 2.1 Materials

CS (including 32% amylose and 68% amylopectin) was supplied by Hebei Gufu Food Co. Ltd., containing 12.9% moisture, 0.25% ash, 0.17% protein, and 0.16% lipid. NC (Cas: 9004‐34‐6), prepared from the cotton grass, was obtained from Shanghai Macklin Biochemical Co. Ltd. α‐amylase (≥ 5000 U/g), derived from porcine pancreas, was purchased from Beijing Psaitong Biotechnology Co. Ltd. (Beijing, China). α‐glucosidase (≥ 50 U/mg) was brought from Shanghai Yuanye Bio‐Technology Co. Ltd. (Shanghai, China). Glucose assay kit was acquired from Nanjing Jiancheng Bioengineering Institute. All other chemical reagents were of analytical grade.

### 2.2 Characterization of NC

The morphology of NC was characterized by scanning electron microscopy (SEM, Sigma300, Zeiss, Germany) following the procedure of Chen et al. (2026). According to the method of Birhanu et al. (2026), the particle size distribution and zeta potential were evaluated using a Zetasizer Nano ZS (Malvern Panalytical, UK).

### 2.3 Preparation of NC‐CS Complexes

Following the method of Yu et al. (2023) with modifications, NC‐CS complexes were prepared. Briefly, the starch suspension (5%, w/v) was mixed with NC at different concentrations (0%, 2.5%, 5.0%, and 7.5% based on the dry weight of starch). The mixture was heated at 95°C for 20 min under continuous stirring, then cooled to 37°C. The resulting samples were freeze‐dried using a vacuum freeze‐dryer, ground into powder, and stored in sealed bags for further use. The CS with 0% NC was the CK, and the other samples were labeled as CS‐2.5% NC, CS‐5.0% NC, and CS‐7.5% NC.

### 2.4 Fourier Transform Infrared Spectroscopy (FTIR)

The starch powder was mixed with dried KBr at a ratio of 1:100 (w/w) and then compressed into pellets. The absorption spectra of different samples were obtained using the following parameters: 4000–400 cm^−1^, a resolution of 4 cm^−1^, 64 scans, and the dried KBr used as the background (Li et al. 2023).

### 2.5 X‐Ray Diffraction (XRD)

The crystal structure of NC‐CS complexes and NC was determined using the XRD (D8, Bruker Inc., USA). The test conditions were as follows: voltage 40 kV, current 40 mA, scanning range 5°–40° (2θ), and scanning rate 4°/min (Xiong et al. 2024).

### 2.6 Microstructural Analysis

The microstructure of NC‐CS complexes was observed using a SEM (Thermo, Holland) (Wang et al. 2025). The starch powder was placed onto the conductive adhesive, followed by spraying with gold. SEM micrographs were taken at 300×.

### 2.7 Solubility (S) and Swelling Powder (SP)

According to the procedure of Yu et al. (2026) with slightly modifications, the S and SP were detected as follows. In brief, the powder (W_1_, dry weight) was mixed with ultrapure water, and the concentration of starch suspension was 1.5%. After being heated in a water bath at 85°C for 30 min with continuous shaking, the samples were cooled to room temperature and then centrifuged at 3200 g for 10 min. The supernatant was collected using a pre‐dried beaker and dried at 105°C for 3 h (W_2_). The remaining sediment was weighted (W_3_). The S and SP were calculated using the following equations:

$$S\;\left(\%\right)=\left(W_{2}/W_{1}\right)\times 100$$

$$\mathrm{SP}\;\left(g/g\right)=W_{3}/\left(W_{1}-W_{2}\right)$$

### 2.8 Transmittance

The starch suspension (1.0%, W/V) was heated at 100°C for 30 min, followed by cooling to room temperature. Absorbance (A) at 620 nm was measured, and the transmittance was calculated using Equation:

$$\text{Transmittance}\;\left(\%\right)=10^{-A}\times 100$$

### 2.9 Differential Scanning Calorimetry (DSC)

The thermal properties of the NC‐CS complexes were analyzed using a DSC3 (Mettler‐Toledo International Inc., Switzerland). The powder (4 mg) and 12 μL of deionized water were mixed in a 40 μL aluminum pan, sealed and then equilibrated at room temperature for 24 h. The test parameters were set as follows: the temperature was increased from 30°C to 160°C at a rate of 10°C/min, and the nitrogen flow rate was 50 mL/min (Yan et al. 2025). The onset temperature (To), peak temperature (Tp), conclusion temperature (Tc), and gelatinization enthalpy (ΔH) of the dsamples were analyzed.

### 2.10 In Vitro Digestibility

According to the method of Wang et al. (2025) with some modifications, the digestibility of NC‐CS complexes was determined. Fresh amylase solution was prepared by suspending 1.00 g α‐amylase (417 U/mL) and 3 mg α‐glucosidase (8.5 U/mL) in 12 mL deionized water, followed by magnetic stirring (300 rpm, 37°C, 10 min) and centrifugation (1400× g, 10 min). 40 mg sample and 4 mL sodium acetate buffer (0.1 M, pH 5.2) were mixed in a cube at 37°C with continuous stirring until the sample was dissolved. 0.5 mL of fresh enzyme solution was added and stirred at 37°C. 100 μL of digestive solution was taken at 0, 20, 30, 60, 90, 120, and 180 min, respectively, and then mixed with 0.1 mL of 95% ethanol to inactivate the enzymes. The mixture was centrifuged (5000 rpm, 6 min), and the supernatant was collected. The glucose was determined using the glucose assay kit (Nanjing Jiancheng Bioengineering Institute, China). The starch digestion ratio was measured according to the following equation:

$$\mathrm{DS}\;\left(\%\right)=\mathrm{Gx}\times 0.9/m\times 100$$

where G_X_: the glucose content at different time, g; m: the mass of the sample, g; DS: the digestion rate.

Rapidly digestible starch (RDS), slowly digestible starch (SDS), and resistant starch (RS) were calculated using the following equations:

$$\mathrm{RDS}\;\left(\%\right)=\left(G_{20}-\mathrm{FG}\right)\times 0.9/T\times 100$$

$$\mathrm{SDS}\;\left(\%\right)=\left(G_{120}-G_{20}\right)\times 0.9/T\times 100$$

$$\mathrm{RS}\;\left(\%\right)=100-\left(\mathrm{RDS}+\mathrm{SDS}\right)$$

where G_20_: the content of glucose at 20 min; G_120_: the content of glucose at 120 min; T: the total weight of starch; FG: the content of free glucose.

The hydrolysis index (HI) was determined according to the method of Yu et al. (2026) using the following equation:

$$\mathrm{HI}\;\left(\%\right)={\mathrm{AUC}}_{\text{sample}}/{\mathrm{AUC}}_{\text{white bread}}\times 100$$

where AUC: the area under the hydrolysis curve.

The estimated GI (eGI) was calculated as follows:

eGI=0.862HI+8.1981

### 2.11 Statistical Analysis

All tests in this study were conducted three times. Data were expressed as the mean ± standard deviation (SD). The data were analyzed by one‐way analysis of variance (ANOVA), followed by Duncan's test in DPS 7.05 (Hangzhou Ruifeng Information Technology Co. Ltd., China). Differences were considered statistically significant at *p* < 0.05.

## 3 Results and Discussion

### 3.1 Characterization of NC

**FIGURE 1:**
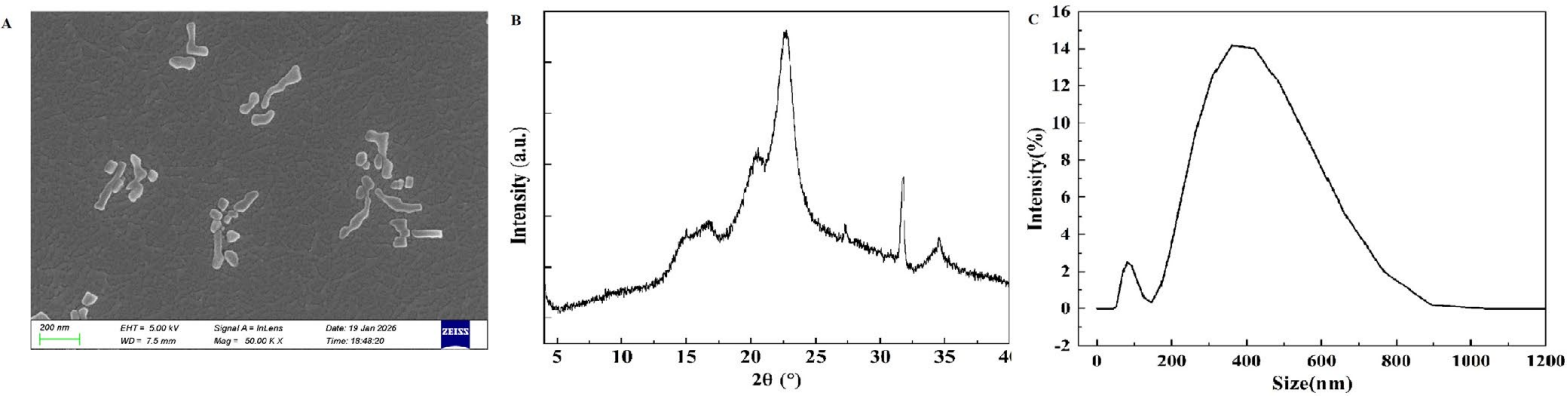
The SEM (A), XRD (B), and particle size distribution (C) of NC. NC, nanocellulose; SEM, scanning electron microscopy; XRD: x‐ray diffraction.

The particle size distribution, XRD, and SEM of NC are presented in Figure 1. The NC showed an irregular shape with diameters of 4–20 nm and an average length of 409 nm. The particle size of nanocellulose ranged from 50 to 894 nm, and the average zeta potential was −37.63 mV. According to Bhattacharjee (2016), nanoparticles with absolute zeta potential values of > 30 mV were highly stable. Therefore, the NC was stable. Moreover, the crystallinity of NC was 82%, and the XRD pattern displayed sharp and intense diffraction peaks at 16.7°, 22.78°, 31.74°, and 34.6° (2θ), corresponding to the crystallographic planes of 101, 002, 110, and 004, respectively. This indicated that the NC retained the crystal structure of natural cellulose I (Liu et al. 2025). Chen et al. (2026) found that carboxylated cellulose nanofiber 1, carboxylated cellulose nanofiber 2, and cellulose nanocrystals exhibited a needle‐like or short rod‐like morphology, with lengths of 168–214 nm. Liu et al. (2025) demonstrated that NC extracted from waste chestnut shells exhibited a rod‐like or entangled fibrous structure.

### 3.2 FTIR of NC‐CS Complexes

**FIGURE 2:**
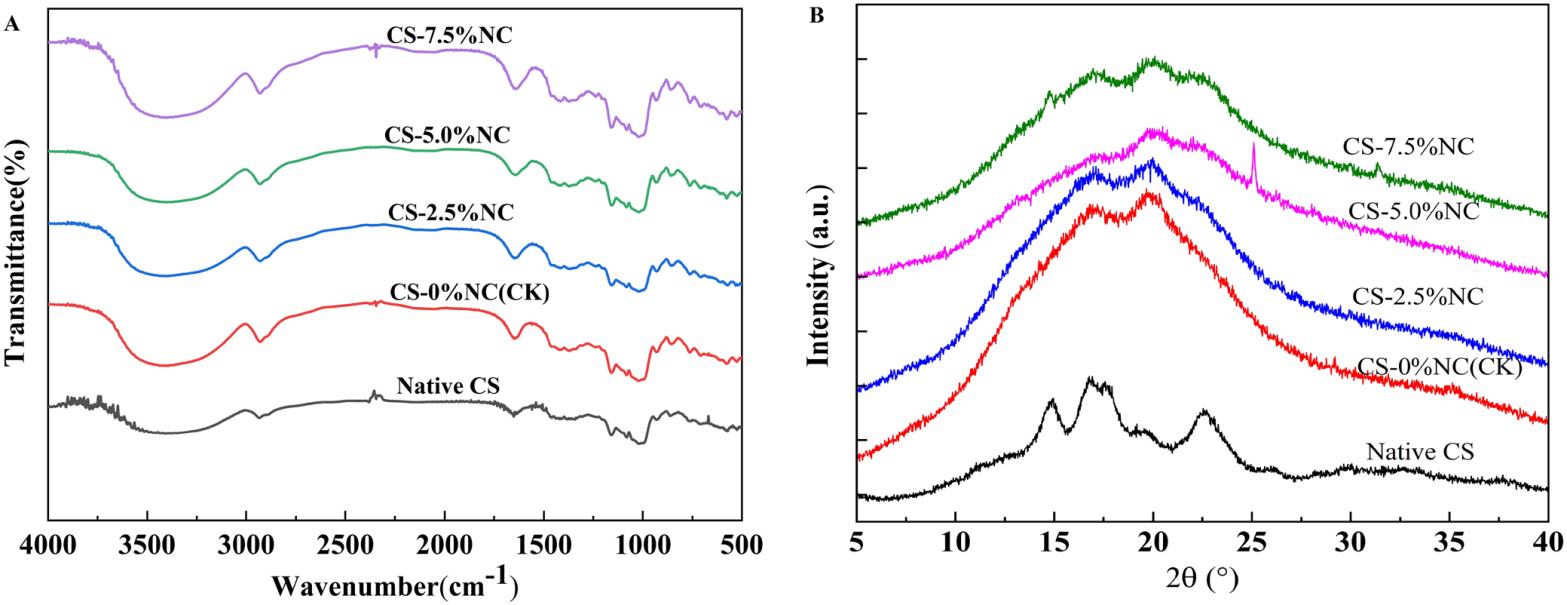
FTIR (A) and XRD (B) spectra of CS with/without NC. CS, corn starch; FTIR, fourier transform infrared spectroscopy; NC, nanocellulose; XRD, x‐ray diffraction.

The FTIR spectra of the native CS and the complexes of NC‐CS are displayed in Figure 2A. All samples exhibited the typical carbohydrate absorption peaks in the range of 4000–400 cm^−1^. A broad band at 3000–3400 cm^−1^ corresponds to O–H stretching, the peak at 2929 cm^−1^ represents the C–H stretching vibrations and the asymmetric stretching of CH_2_, and the peak at 1154 cm^−1^ is due to the C–O bond stretching (Cen et al. 2025). Compared with the native starch, all the complexes showed no new peaks, indicating that NC combined to the CS through non‐covalent bonds. Moreover, the wavenumbers of –OH peak in native and CK (gelatinized CS) were 3409 cm^−1^ and 3400 cm^−1^, whereas the –OH peaks in NC‐CS complexes moved to lower wavenumbers, ranging from 3400 cm^−1^ to 3370 cm^−1^ (Table 1). Also, the intensity of NC‐CS complexes at 3600–3000 cm^−1^ increased as the NC content increased. It implied that hydrogen bonds were formed between the CS and NC.

**TABLE 1:** Results of FTIR deconvolution, crystallinity, and DSC for NC‐CS complexes.

Sample	FTIR		Relative crystallinity (%)	DSC			
	Shift_3290_	R_1047/1022_		To (°C)	Tp(°C)	Tc (°C)	ΔH (J/g)
CK	3400 ± 1.58^a^	0.38 ± 0.04^b^	28.30 ± 0.14^b^	97.26 ± 0.64^b^	107.12 ± 0.66^b^	121.80 ± 2.11^b^	5.01 ± 0.92^a^
CS‐2.5% NC	3400 ± 2.36^a^	0.70 ± 0.03^a^	26.75 ± 0.35^c^	99.22 ± 0.11^a^	108.46 ± 0.52^ab^	122.6 ± 1.33^ab^	5.00 ± 0.49^a^
CS‐5.0% NC	3392 ± 3.69^b^	0.71 ± 0.03^a^	28.65 ± 0.21^ab^	99.21 ± 0.14^a^	113.32 ± 1.7^a^	125.06 ± 4.35^a^	4.48 ± 0.60^a^
CS‐7.5% NC	3370 ± 2.45^c^	0.72 ± 0.02^a^	29.05 ± 0.07^a^	99.75 ± 0.47^a^	113.72 ± 1.3^a^	132.31 ± 4.87^a^	4.99 ± 0.50^a^

Abbreviations: CS, corn starch; DSC, differential scanning calorimetry; FTIR, Fourier transform infrared spectroscopy; NC, nanocellulose; Tc, conclusion temperature; To, onset temperature; Tp, peak temperature; ΔH, gelatinization enthalpy. Values of means followed by different lowercase letters in the same column are significantly different (*p* < 0.05).

The spectrum in the range of 1200–800 cm^−1^ was used to analyze the short‐range ordered structure of starch. The peak at 1047 cm^−1^ represents the crystalline and ordered regions, while the characteristic absorption peak at 1022 cm^−1^ is attributed to the amorphous fraction of starch. The ratio of 1047/1022 cm^−1^ (R_1047/1022_) was used to characterize the short‐range ordered structure in the crystalline regions. As shown in Table 1, the R_1047/1022_ of gelatinized CS (CK) was 0.384, whereas the R_1047/1022_ values for the NC‐CS complexes were 0.7–0.715, suggesting a significant enhancement in structural orderliness. Cen et al. (2025) showed that the fermented soluble dietary fiber extracted from sweet potato residue enhanced the values of R_1047/1022_ of wheat starch. Chen et al. (2026) reported that cellulose nanocrystals and carboxylated cellulose nanofiber promoted the formation of the short‐range order of rice starch.

### 3.3 XRD of NC‐CS Complexes

Figure 2 displays the XRD pattern of the native CS and the complexes of CS and NC. The XRD pattern of native CS revealed an A‐type crystalline structure, characterized by distinct peaks at 2θ = 14.82°, 16.80°, 17.72°, and 22.48°. After gelatinization, peaks (14.82°, 17.72°, and 22.48°) disappeared, and all samples displayed only two diffraction peaks (17.72° and 19.70°), indicating that an A + V‐type crystalline structure was formed. The relative crystallinity (RC) of CK (gelatinized CS) was 28.3%, while the RC of the NC‐CS complexes ranged from 26.75% to 29.05%. Luo et al. (2025) observed that maize starch‐dietary fiber complexes were the A + V crystal type. Xiong et al. (2024) found that the complexes of soluble dietary fiber from pomegranate peel with sweet potato starch exhibited a B‐type crystalline structure.

### 3.4 SEM of NC‐CS Complexes

**FIGURE 3:**
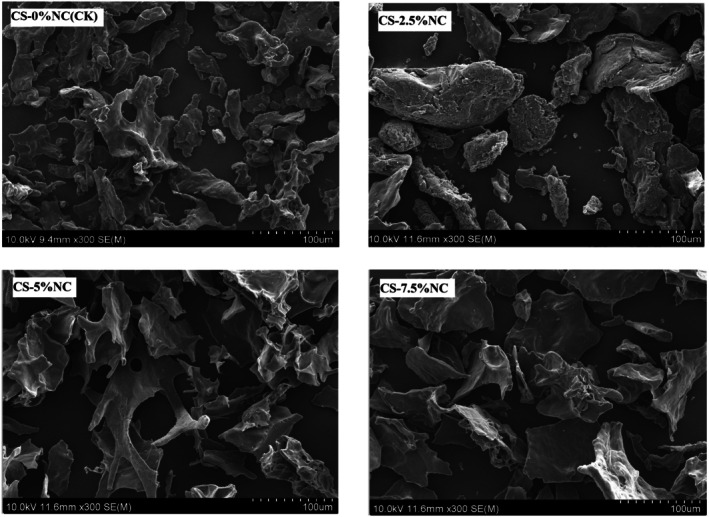
The microstructure of NC‐CS complexes. CS, corn starch; NC, nanocellulose.

The SEM images of the NC‐CS complexes are shown in Figure 3. After gelatinization, the CS exhibited small, thick fractured particles with rough surfaces. The complexes with 2.5% NC showed numerous aggregates with irregular shapes. With the increase of NC, the complexes became thinner and smoother. This can be attributed to the hydroxyl groups (‐OH) on the surface of NC, which formed strong hydrogen bonds with the hydroxyl groups of amylose and amylopectin. As the NC addition increased, an extensive hydrogen‐bonding network was formed. This network restricted their rearrangement and migration during the cooling process, thereby resulting in a more uniform, smooth, and compact structure. Lv et al. (2025) found that the cassava starch‐cellulose complexes showed a smooth, irregular morphology, which was consistent with our observations at higher NC levels. Wei et al. (2023) demonstrated that laminarin‐wheat starch mixtures were thicker and rougher aggregates, suggesting that the specific polysaccharide structure and interaction affinity may influence the final morphology.

### 3.5 DSC of NC‐CS Complexes

As shown in Table 1, the To, Tp, and Tc of CK were 97.26°C, 107.12°C, and 121.80°C, respectively. After mixing with the NC, the To, Tp, and Tc of the complexes ranged from 99.22°C to 99.75°C, 108.46°C to 113.72°C, and 121.80°C to 132.31°C, respectively. The enhancement of the To, Tp, and Tc may be due to the NC that can absorb water and form a barrier on the starch surface, thereby slowing water penetration into the granules. Moreover, no significant difference in ΔH was observed between CK and the NC‐CS complexes. Ji et al. (2025) revealed that inulin increased the To, Tp, and Tc and decreased the ΔH of heat‐moisture treated wheat starch. Fang et al. (2025) showed that insoluble dietary fiber reduced ΔH and increased To, Tp, and Tc of wheat starch.

### 3.6 S, SP, and Transmittance

**FIGURE 4:**
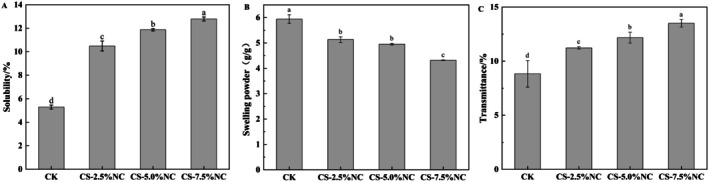
The S, SP, and transmittance of the NC‐CS complexes. CS, corn starch; NC, nanocellulose; S, solubility; SP, swelling powder.

Figure 4 displays the S, SP, and transmittance of the NC‐CS complexes. The S of the CK was 5.29%. With increasing NC content, the S of the complexes increased significantly, ranging from 10.49% to 12.79%, indicating that NC promoted the CS dissolution. Moreover, the SP of the complexes exhibited a decreasing trend with rising NC concentration. This may be due to the NC adhering to the surface of the starch granules, thereby restricting the starch's swelling and gelatinization during heating. Many studies have reported that the SP of starch was reduced by the addition of polysaccharides (Shen et al. 2025; Zhang et al. 2024). However, Yan et al. (2025) found that flaxseed gum improved the SP of wheat starch, suggesting that the effect depends on the type and structure of the polysaccharide.

High transparency serves as an essential criterion for the processing of foods such as jelly and starch noodles. With the addition of NC, the transmittance of the NC‐CS complexes increased, and there was a significant difference between CK and the NC‐CS complexes. It was due to that NC restricted the swelling of starch and led to the formation of small, uniform granules. Zheng et al. (2026) found that the transparency of both citric acid‐modified starch and malic acid‐modified starch increased significantly.

### 3.7 In Vitro Digestibility

The contents of RDS, SDS, and RS in the NC‐CS complexes are displayed in Table 2. Compared to CK, as the NC content increased, RDS decreased from 41.26% to 30.27%, SDS increased from 32.01% to 58.07%, RS decreased from 26.73% to 11.66%, and the SDS + RS increased from 58.74% to 69.73%. This was attributed to the NC, which interacted with starch molecules via hydrogen bonds, thereby blocking or masking the active sites of amylase. Furthermore, the eGI of CK was 67.69, whereas that of the NC‐CS complexes ranged from 64.92 to 53.69. Overall, NC decreased the digestibility of CS and increased the content of SDS + RS and SDS. Xiong et al. (2024) found that soluble dietary fiber from pomegranate peel increased the RS content in sweet potato starch. Cen et al. (2025) demonstrated that the fermented soluble dietary fiber extracted from sweet potato residue increased RS and SDS and decreased the digestibility rate. Li et al. (2025) showed that modified okara dietary fiber increased the RS and SDS + RS content in wheat starch. In addition, it is important to note that the in vitro digestion method does not fully simulate physiological digestion. Therefore, in vivo digestion would be conducted in future.

**TABLE 2:** The in vitro digestibility of NC‐CS complexes.

Samples	RDS (%)	SDS (%)	RS (%)	SDS + RS (%)	HI (%)	eGI
CK	41.26 ± 0.81^a^	32.01 ± 3.42^c^	26.73 ± 2.62^a^	58.74 ± 0.81^b^	69.02 ± 0.90^a^	67.69 ± 0.78^a^
CS‐2.5% NC	30.61 ± 0.37^b^	47.99 ± 1.83^b^	21.41 ± 1.47^b^	69.39 ± 0.37^a^	65.80 ± 0.25^b^	64.92 ± 0.22^b^
CS‐5.0% NC	30.27 ± 0.19^b^	58.07 ± 0.38^a^	11.66 ± 0.58^c^	69.73 ± 0.19^a^	61.18 ± 0.18^c^	60.94 ± 0.15^c^
CS‐7.5% NC	31.11 ± 0.21^b^	48.64 ± 1.03^b^	20.26 ± 1.24^b^	68.89 ± 0.21^a^	52.78 ± 0.15^d^	53.69 ± 0.13^d^

Abbreviations: CS, corn starch; eGI, the estimated glycemic index; HI, the hydrolysis index; NC, nanocellulose; RDS, rapidly digestible starch; RS, resistant starch; SDS, slowly digestible starch.Values of means followed by different lowercase letters in the same column are significantly different (*p* < 0.05).

## 4 Conclusion

This study investigated the effects of NC on the physicochemical properties, multi‐scale structure, and in vitro digestibility of CS. Results showed that NC interacted with CS primarily through non‐covalent bonds, leading to an enhancement in short‐range molecular order. XRD revealed that the NC‐CS complexes formed an A + V‐type crystalline structure, with relative crystallinity ranging from 26.75% to 29.05%. SEM showed that the NC‐CS complexes exhibited thin and smooth surfaces. DSC demonstrated that the To, Tp, and Tc of CS increased upon complexation with NC, whereas the ΔH remained unchanged. Furthermore, the incorporation of NC increased the S and transparency but decreased the SP of CS. The SDS + RS increased, whereas RDS decreased. The SDS was enhanced with the highest SDS content at 5% NC. In all, NC affected the physicochemical properties and multi‐scale structure of CS, thereby reducing the in vitro digestibility. Quantitative interaction analysis (e.g., NMR, molecular docking) and in vivo digestibility would be performed in the future. This study provides a foundation for the application of NC to develop healthy starch‐based foods.

## Author Contributions

**Yanyan Liu:** writing – original draft, writing – review and editing, methodology, conceptualization, investigation, data curation. **Shenghua He:** methodology. **Huiying Zhang:** investigation, data curation. **Shuyao Li:** investigation, data curation. **Junjie Yuan:** investigation, data curation. **Yonghui Wang:** conceptualization, methodology, validation. **Weiyun Guo:** visualization, resources. **Guanghui Li:** funding acquisition, methodology, supervision. **Xueli Gao:** project administration, software, funding acquisition, methodology, supervision.

## Funding

This work was supported by Teachers Key Project in Universities of Henan Province, 2024GGJS120. Natural Science Foundation of Henan Province, 262300420085. Research‐based Teaching Project in Universities of Henan Province, 2023‐388‐120. Open project of Collaborative Innovation Center of Functional Food by Green Manufacturing, Henan Province, 2024XTKF024. Innovation and Entrepreneurship Training Program for University Students in Xuchang University, X202610.

## Conflicts of Interest

The authors declare no conflicts of interest.

## Data Availability

The data that support the findings of this study are available on request from the corresponding author. The data are not publicly available due to privacy or ethical restrictions.
